# Cancer immunotherapy by silencing transcription factor c-Rel using peptide-based nanoparticles

**DOI:** 10.3389/fimmu.2025.1554496

**Published:** 2025-03-11

**Authors:** Shuyao Lang, Yuxuan Zhu, Zibin Tan, Yu Zhang, Ruijing Liang, Jian Ren, Ping Li, Fan Pan, Lintao Cai, Youhai H. Chen

**Affiliations:** ^1^ Center for Cancer Immunology, Shenzhen Institutes of Advanced Technology, Chinese Academy of Sciences, Shenzhen, China; ^2^ Key Laboratory of Cellular and Gene Therapy of Guangdong Province, Faculty of Pharmaceutical Sciences, Shenzhen University of Advanced Technology, Shenzhen, China; ^3^ Guangdong Key Laboratory of Nanomedicine, CAS-HK Joint Lab for Biomaterials, Shenzhen Institutes of Advanced Technology, Chinese Academy of Sciences, Shenzhen, China; ^4^ Sino-European Center of Biomedicine and Health, Shenzhen, China

**Keywords:** cancer immunotherapy, immune checkpoint, myeloid-derived suppressor cells, c-Rel, NF-κB, tumor microenvironment

## Abstract

**Background:**

Cancer immunotherapy has shown promising results in the clinic, but it faces great challenges such as low response rates and low efficacy in solid tumors. c-Rel, a member of the nuclear factor (NF)-κB family, is a newly described immune checkpoint for myeloid-derived suppressor cells (MDSCs), which contribute to the formation of immune-suppressive tumor microenvironment and resistance to cancer immunotherapy. How to selectively target myeloid c-Rel for the treatment of cancer is not well established. In this study, we investigated the feasibility and efficacy of knocking down myeloid c-Rel with siRNA-loaded peptide-based nanoparticles as a new cancer immunotherapy strategy.

**Methods:**

The knockdown of c-Rel gene by the siRNA-loaded peptide nanoparticles was confirmed on MDSCs *in vitro* and *in vivo*. The effects of c-Rel silencing on cell number and immune suppressive function of the murine bone marrow-derived MDSCs were then investigated. To evaluate the anti-tumor efficacy of the c-Rel siRNA loaded nanoparticles, female C57BL/6 mice with subcutaneous B16 tumor were treated with PBS, c-Rel siRNA loaded nanoparticles, control siRNA loaded nanoparticles or empty nanoparticles. The tumor growth and body weight of mice were monitored, and the numbers and immune activities of tumor infiltrated immune cells in different groups were analyzed at the end of the experiment. The immune function of MDSCs isolated from tumor bearing mice received different treatments were further investigated *ex vivo* by T cell proliferation assays.

**Results:**

The c-Rel siRNA nanoparticles significantly reduced c-Rel expression in MDSCs, diminished both the number and immune suppressive function of MDSCs, and enhanced intratumor CD8^+^ T cell responses. Significantly reduced tumor growth was observed in mice treated with the c-Rel siRNA nanoparticles compared to control mice.

**Conclusion:**

Our data indicates that peptide-based nanoparticles can be successfully utilized to target the myeloid immune checkpoint c-Rel for the treatment of cancer.

## 1 Introduction

Cancer immunotherapy has become the “fourth pillar” of cancer therapy, benefiting many of patients with cancer (1, 2). However, the response rate and therapeutic efficacy of cancer immunotherapy in solid tumors still remain unsatisfactory (1, 3–5). One of the major challenges of immunotherapy in solid tumors is the immune-suppressive tumor microenvironment (TME), which helps tumor cells to evade immune surveillance and prevents the generation of anti-tumor immune responses (1, 6–8). Myeloid-derived suppressor cells (MDSCs) are a type of heterogeneous immune suppressor cells which play a key role in the formation of immune-suppressive microenvironment (9–12). In mice, MDSCs are usually described as Gr-1^+^CD11b^+^ cells, which consist of two major subpopulations, M-MDSCs and PMN-MDSCs. MDSCs support the tumor growth and immune evasion via multiple mechanisms. They down-regulate the proliferation and activity of anti-tumor T cells and NK cells, express multiple immune suppressive molecules such as IL-10, Arg-1, iNOS and PD-1, and induce the generation of regulatory T cells (T_reg_). MDSCs are rare in healthy people, but the population can significantly expand in cancer patients. It has been reported that the numbers of MDSCs in cancer patients negatively correlate with clinical prognosis and the response rate of immunotherapy (10, 13–16). Several clinical trials aimed at reducing the number or function of MDSCs have reported positive results (11, 13, 17).

The transcription factor c-Rel, encoded by the *Rel* gene, is one of the five members of the NF-κB family and has been known to be a risk factor of cancer and inflammatory diseases (18). Our recent study suggested that c-Rel is also a myeloid immune checkpoint which is crucial for the development and function of MDSCs (19, 20). The knockout of *Rel* in myeloid cells significantly reduced the number of both M-MDSC and PMN-MDSC subpopulations and reduced tumor growth in mice. Unlike other ubiquitously expressed NF-κB family members, c-Rel is preferentially expressed by myeloid and lymphoid cells. Therefore, drugs targeting c-Rel are expected to have low systematic toxicity. Though several research has reported that the down-regulation of c-Rel can treat inflammatory diseases such as psoriasis and arthritis (21, 22), the therapeutic effect of c-Rel gene knockdown in cancer treatment is less explored. We hypothesize that knocking down c-Rel gene *in vivo* can reduce the immune suppressive microenvironment by diminishing the number or suppressive function of MDSCs in tumor, thus enhancing anti-tumor immunity and reducing tumor growth. Small interfering RNA (siRNA) is a convenient method to knockdown genes such as c-Rel *in vivo*. In this study, we utilized biodegradable poly(amino acid) nanoparticles (NPs) to deliver c-Rel siRNA *in vivo*, which led to significant c-Rel silencing and substantially slowed tumor growth.

## 2 Materials and methods

### 2.1 Animals

6 to 10-week-old female C57BL/6 mice were used in the experiments and kept under pathogen-free conditions at the animal core facility of the Shenzhen Institutes of Advanced Technology, Chinese Academy of Sciences. All procedures were pre-approved by the Institutional Animal Care and Use Committee (SIAT-IACUC-20240305-YYS-AZZX-LSY-01).

### 2.2 Cells

B16F10 and RAW cells were cultured in Dulbecco’s modified Eagle medium (DMEM) supplemented with 2 mM L-glutamine, 10% Fetal Bovine Serum (FBS) and 1% penicillin-streptomycin (100 U/ml penicillin and 100 μg/ ml streptomycin). Murine bone marrow-derived MDSCs were cultured in Roswell Park Memorial Institute (RPMI) medium supplemented with 10% FBS, 1% penicillin-streptomycin, GM-CSF (100 ng/ml) and IL-6 (100 ng/ml). All cultures were maintained in a humidified 5% CO_2_ incubator at 37°C.

### 2.3 Reagents

DNase/RNase free water, RNA easy isolation reagent, ChamQ SYBR qPCR Master Mix, HiScript^®^ II Q RT SuperMix for qPCR (+gDNA wiper) were purchased from Vazyme; mPEG_45_-PLL_30_-PLLeu_40_ was purchased from Xi’an Ruixi Chemical Company; FBS, GelStain Blue were purchased from Transgen Biotech; DMEM, RPMI, sodium pyruvate, N-2-hydroxyethylpiperazine-N-2-ethane sulfonic acid buffer (HEPES), penicillin-streptomycin, collagenase type IV were purchased from Gibico; GM-CSF and IL-6 were purchased from PeproTech; PE-anti-mouse c-Rel antibody (1RELAH5), cell stimulation cocktail (500X) were purchased from eBioscience; APC-anti-mouse Gr-1, FITC-anti-mouse/human CD11b, BV510-anti mouse CD45, BV510-anti mouse CD8, FITC anti-mouse CD4, APC/cy7 anti mouse IFN-γ, PerCP anti-mouse CD4, PerCP anti mouse PD-1, PE anti-mouse CD69, PerCP anti-mouse CD107a, APC anti-mouse FoxP3 were purchased from Biolegend; Purified anti-mouse c-Rel antibody and HRP-Goat anti-mouse antibody for Western detection was purchased from Santa Cruz Biotechnology; 5(6)-Carboxyfluorescein diacetate succinimidyl ester (CFSE) and Bovine serum albumin (BSA) were purchased from Yeason Biotechnology (Shanghai); Radioimmunoprecipitation assay (RIPA) and buffer Phosphate Buffered Saline (PBS) were purchased from Beyotime Biotechnology; Polyvinylidene fluoride (PVDF) membrane, Lipopolysaccharide (LPS) and DNase I were purchased from Millipore-Sigma; Super ECL Detection Reagent was purchased from Thermo Scientific Fisher; LIVE/DEAD violet viability kit, Ethylenediaminetetraacetic acid (EDTA), Intracellular Fixation & Permeabilization Buffer Set and FOXP3/Transcription Factor Staining Buffer Set were purchased from Invitrogen; EasySep™ Mouse MDSC (CD11b^+^Gr1^+^) Isolation Kit was purchased from StemCell Techonologies; Targeting mouse c-Rel siRNA (sense: 5’-CAACCGGACAUACCCGUdTdT-3’, anti-sense: 5’-AGACGGGUAUGUCCGGUUGdTdT-3’) and negative control siRNA (sense: 5’-UUCUCCGAACGUGUCACGUdTdT-3’, anti-sense: 5’-ACGUGACACGUUCGGAGAAdTdT-3’) were supplied by Shanghai GenePharma Co. Ltd.

### 2.4 Preparation of siRNA-loading polymer nanoparticles

mPEG_45_-PLL_30_-PLLeu_40_ (23) was dissolved with DNase/RNase-free water (10 mg/ml) and set on a shaker overnight to allow the self-assembly of nanoparticles. The resulting stock solution is stable at room temperature for two months. siRNA loading was done by pipette mixing of siRNA stock solution (50 pmol/μl in water) and NP stock solution (10 mg/ml in water) at N/P ratio = 5 (siRNA: NP = 2.11: 1 v/v), then stand at room temperature for 30 min. The resulting solution of siRNA loaded NPs was adjusted with 10x PBS and 1x PBS to a final concentration of 20 pmol/μl in 1x PBS for further study. The siRNA loading was estimated via electrophoresis (2% (w/v) agarose gel containing 1x GelStain Blue,15 min at 40 V in TAE buffer). The sizes and zeta potentials of the self-assembled NPs were determined by Zetasizer Nano ZS (Malvern Instrument).

### 2.5 Generation of MDSCs from murine bone marrow

Murine bone marrow was collected from naïve wild type (WT) C57BL/6 mice aged 8-10 weeks and cultured in complete RPMI medium containing GM-CSF (100 ng/ml) and IL-6 (100 ng/ml) at a density of 1 x 10^6^/ml for 5 days to induce MDSC development. For each independent experiment, bone marrow cells were collected and pooled from two mice. At day 3, half volume of fresh culture medium was added to the culture to maintain cell growth. The bone marrow derived MDSCs were collected at day 5 of culture for study. The purity of MDSCs was analyzed by flow cytometry after cell surface staining of Gr-1 and CD11b (Gr-1^+^CD11b^+^). Cell counting beads were added for flow cytometry analysis of the absolute count of each MDSC sample.

### 2.6 *In vitro* siRNA transfection

RAW cells (0.4x10^6^/well) or murine MDSCs (0.8x10^6^/well) were seeded in 12-well plate and cultured overnight before transfection. Free c-Rel siRNA, c-Rel siRNA loaded NPs (NP_c-Rel_), control siRNA loaded NPs (NP_NC_) or empty NPs (NP_empty_) were added to the cell culture to a final siRNA concentration of 100 pmol/ml (NP concentration = 9.45 μg/ml). PBS was used as vehicle control. For qPCR detection of c-Rel mRNA expression, cells were co-cultured with different treatments for 6 or 16 hours, then washed with PBS and collected for RNA extraction and qPCR analysis. For Western blot detection of c-Rel protein, LPS (50 ng/ml final concentration) was added to all samples at the time of treatment addition in order to elevate the c-Rel expression level for a better detection, then the cells were co-cultured with LPS and different treatments for 24 h before collecting for protein extraction and Western blot detection.

For *in vitro* T cell suppression assay, murine bone marrow cells (1 x 10^6^/ml, 3 ml/well) were cultured in 6-well plates with complete RPMI medium containing GM-CSF (100 ng/ml) and IL-6 (100 ng/ml) to induce the development of MDSCs. PBS, NP_c-Rel_ or NP_NC_ (final siRNA concentration = 100 pmol/ml) were added at day 0, 2 and 4 of culture. The *ex vivo* induced MDSCs with different treatments were collected on day 5 for *in vitro* T cell suppression assay.

### 2.7 c-Rel knockdown selectivity in splenocytes

To determine c-Rel knockdown selectivity, spleens were isolated form mice (n = 3) and meshed into single cell suspension, after red blood cell lysis, splenocytes were washed with PBS and seeded into 12 well plate (0.8x10^6^/well), followed by the addition of NP_c-Rel_, NP_NC_, or NP_empty_ to a final siRNA concentration of 100 pmol/ml. PBS was used as vehicle control. Splenocytes were incubated with the indicated treatments for 24 h, then collected by centrifuge and washed with PBS twice. The c-Rel knockdown selectivity in different cell types (CD11b^+^, CD4^+^ and CD8^+^) were analyzed by mean fluorescence intensity (MFI) of PE signal by flow cytometry.

### 2.8 *In vitro* T cell suppression assay

Splenocytes were collected from healthy WT C57BL6 aged 8-10 weeks. After red blood cell lysis, the splenocytes were washed with PBS twice and adjusted to 1 x 10^7^/ml. Final concentration of 1 μM CFSE was added to the cell stock and then kept under 37 degree for 10 min to allow surface labeling. Twice volume of complete medium was then added to the cell mixture and kept at room temperature for 10 min to quench the reaction. The resulting CFSE labeled splenocytes were washed with complete medium for three times, then adjusted to 1x10^6^/ml with complete RPMI medium supplemented with 25 mM HEPES, 55 μM 2-mercaptoethanol, and anti-mouse CD28 (125 ng/ml). The CFSE labeled splenocytes were then seeded to 96-well plate (100 μl/well, containing 1x10^5^ cells) with plate-bounded anti-mouse CD3 (250 ng/ml), and cultured at 37 degree for 2 h before addition of MDSCs. As non-activated control, three wells on the plate were seeded with CFSE labeled splenocytes but without anti-CD3 coating.

*Ex vivo* induced MDSCs or MDSCs isolated from spleens of tumor bearing mice in different treatment groups were collected and washed with complete RPMI twice, then resuspend with 1 ml complete medium. A small fraction of cells (50 μl out of the 1 ml cell stock) were collected and surface stained with anti-Gr-1 and CD11b antibodies, then cell counting beads were added for flow cytometry analyisis of absolute count of Gr-1^+^CD11b^+^ cells. Based on the result of flow cytometry, the cell stock was adjusted to 5x10^5^ Gr-1^+^CD11b^+^ cells/ml with complete medium. The resulting MDSC cell stock was then added to the 96-well plate containing CFSE labeled splenocytes at desired ratio (1:2, 1:4 and 1:8). The final cell culture volume was adjusted to 200 μl/well for all samples with complete RPMI medium. Three wells on the plate were seeded with only CFSE labeled splenocytes but no MDSCs.

The CFSE labeled splenocytes were co-cultured with MDSCs at different ratios for 48 h, followed with surface staining of anti-mouse CD4 and CD8 antibodies. The T cell proliferation suppressions were analyzed by flow cytometry (gated on CD4^+^ and CD8^+^ cells) by the dilution of CFSE fluorescence compared with non-activated T cells. The percentage of T-cell suppression was calculated as follows: ((% of T-cell proliferation of cultures without MDSCs − % of T-cell proliferation of cultures with MDSCs received different treatments)/% of T-cell proliferation of cultures without MDSCs) × 100.

### 2.9 *In vivo* c-Rel silencing

Female WT C57BL/6 mice (8-10 week-old) bearing B16 tumor (300-600 mm^3^, 2 mice/group) were intravenously (i.v.) injected with PBS, NP_c-Rel_ (2000 pmol siRNA/dose), NP_NC_ (2000 pmol siRNA/dose) or NP_empty_ (equal amount of NPs). Blood samples (20-50 μl/mouse) were collected from tail vein 24 h after injection. After red blood cell lysis, the cells were surface stained with anti-mouse Gr-1 and anti-mouse CD11b antibodies, followed by fixation, permeabilization and intracellular staining of PE-anti-mouse c-Rel antibodies. The *in vivo* c-Rel silencing was analyzed by MFI of PE signal on flow cytometry (gated on Gr-1^+^CD11b^+^).

### 2.10 Tumor model and treatments

Female WT C57BL6 mice aged 6-8 weeks were s.c. planted with B16 melanoma cells (1 x 10^5^/mouse) at right flank. Mice were randomly divided into four groups by their initial tumor size on day 7. On day 8, 11, 14 and 17, mice were i.v. injection of PBS, NP_c-Rel_ (2000 pmol siRNA/dose), NP_NC_ (2000 pmol siRNA/dose) or NP_empty_ (equal amount of NPs) through tail vein. Tumor sizes and body weights of mice were measured every other day. Tumor volume was calculated using the formula: V = L x W x H/2 (mm^3^). Three independent experiments were pooled for data presentation. Sample size: PBS (n = 26 mice), NP_empty_ (n = 22 mice), NP_NC_ (n = 25 mice), NP_c-Rel_ (n = 30 mice). Mice with poor body condition or no initial tumor growth were excluded on randomization, no exclusions were made after randomization.

### 2.11 RNA extraction and qPCR

Total RNA was extracted with RNA easy-isolation kit according to the manufacturer’s instructions. Reverse transcription was performed using HiScript^®^ II Q RT SuperMix, followed by quantitative PCR which carried out with the Applied Biosystems Quantstudio 3 using the ChamQ SYBR Green PCR Master Mix. Relative levels of gene expression were determined using GAPDH mRNA as the control. Primer sequences for qPCR are listed in **Table 1**.

**Table 1:** Primer sequences for qPCR.

mouse *Gapdh* Forward	AGTATGACTCCACTCACGGCAA
mouse *Gapdh* Reverse	TCTCGCTCCTGGAAGATGGT
mouse *Rel* Forward	ACAACAACCGGACATGGCC
mouse *Rel* Reverse	GGTCTGCGTTCTGGTCCAA
mouse *Arg1* Forward	GCTCAGGTGAATCGGCCTTTT
mouse *Arg1* Reverse	TGGCTTGCGAGACGTAGAC
mouse *Nos2* Forward	CACCAAGCTGAACTTGAGCG
mouse *Nos2* Reverse	CGTGGCTTTGGGCTCCTC
mouse *Cebpb* Forward	GACAAGCTGAGCGACGAGTA
mouse *Cebpb* Reverse	AGCTGCTCCACCTTCTTCTG

### 2.12 Western blot

RIPA buffer was used to lyze cells (100 μl/well for 6-well plates). Cell lysates were centrifuged at 3000 g for 5 min, then the supernatant was subjected to 12% SDS–polyacrylamide gel electrophoresis (30 min at 70 V, then another 60 min at 120 V) and transferred to PVDF membranes. The membranes were stained overnight at 4°C with antibodies specific for c-Rel, and β-actin, followed by incubation for 1 h at room temperature with secondary antibodies conjugated with peroxidase. Membrane-bound immune complexes were detected with the Super ECL Detection Reagent on GelView 5000ProII Imager (Guangzhou Biolight Biotechnology).

### 2.13 Flow cytometry analysis

To prepare single-cell suspension of tumor samples, tumors were cut into small pieces and digested in complete RPMI 1640 supplemented with 1 mg/ml collagenase type IV and 0.1 mg/ml DNase I for 40 min at 37°C. The resulting mixture was filtered through 70 μm cell strainer followed by three PBS washes to obtain single cell suspension. For spleen and lymph node samples, single-cell suspension of the spleen was obtained by mechanical disintegration in flow cytometry staining buffer followed by 70 μm cell strainer filtration. Cells were washed with PBS twice before staining. To detect IFN-γ expression, cells were first incubated for 6 h with cell stimulation cocktail (1:500 dilution in RPMI), then washed with PBS three times before staining.

For flow cytometry staining, the resulting single cell suspensions were first stained with LIVE/DEAD violet viability kit (1:2000 final dilution in PBS) at room temperature in dark for 10 min. Flow cytometry staining buffer (0.5% BSA+ 1 mM EDTA in PBS) was added to quench the reaction, followed by two additional washing with flow cytometry staining buffer. Antibodies for cellular surface staining were then added to the samples (1:400 dilution in flow cytometry staining buffer), and allowed to stain at 4 degree in dark for 15 min. After washing with flow cytometry staining buffer twice, cells were fixed and permeabilized with FOXP3/Transcription Factor Staining Buffer Set (for c-Rel and FoxP3 staining) or Intracellular Fixation & Permeabilization Buffer Set (for IFN-γstaining) according to the manufacturer’s instructions respectively, followed by addition of intracellular staining antibodies (1:200 dilution). The intracellular staining was performed overnight at 4 degree in dark. Then cells were collected and washed with PBS twice before flow cytometry analysis. The stained cells were analyzed on Attune NxT flow cytometer (Invitrogen by Thermo Fisher Scientific). Data were analyzed with the FlowJo software (FlowJo LLC, version 10.10.0).

### 2.14 Isolation of Gr-1^+^CD11b^+^ cells from the spleens of tumor-bearing mice

The spleens of tumor-bearing mice were collected and processed into single-cell suspension as described above. Without red blood cell lysis, the cell suspension was adjusted to 1 x 10^8^/ml. For each spleen, 0.5 ml of cell suspension (5 x 10^7^ cells) was processed with EasySep™ Mouse MDSC (CD11b^+^Gr1^+^) Isolation Kit following the manufacturer’s instructions. A 20-times enrichment can be achieved after the isolation process. The suppressive function of the enriched Gr-1^+^CD11b^+^ cells were measured by T cell suppression assay.

### 2.15 Statistics

Sample size calculations were based on the tumor sizes observed in the pilot experiments. Power calculations were performed via using Experimental Design Assistant (https://eda.nc3rs.org.uk/eda/login/auth) with >80% power at the 0.05 significance level (>= 4 mice per group). Statistical analyses were performed using Prism 10.0.3 (GraphPad Software). For comparisons between two groups, a two-tailed unpaired t-test was used. Mice were randomly assigned into experimental groups for *in vivo* studies. No data were excluded from the analyses. Bars represent mean ± standard error of mean (SEM). The investigators were not blinded for the experiments and data analyses.

## 3 Results

### 3.1 c-Rel siRNA-loaded nanoparticles (NP_c-Rel_) effectively silenced c-Rel expression *in vitro* and in mice

Free siRNAs have a low cell permeability and a short lifetime *in vivo*. In this study, we prepared degradable polypeptide micelle nanoparticles of a triblock copolymer, i.e., Poly(ethylene glycol)_45_-b-poly(l-lysine)_30_-b-poly(l-leucine)_40_ (mPEG_45_–PLL_30_–PLLeu_40_) (23), for myeloid c-Rel silencing (**Supplementary Figure 1A**). mPEG_45_–PLL_30_–PLLeu_40_ can self-assemble into nanoparticles with an average size of 100 nm in water. To load the c-Rel siRNA or negative control (NC) siRNA, the mPEG_45_–PLL_30_–PLLeu_40_ nanoparticles were gently mixed with siRNA solution at desired N/P ratio, followed by standing for 30 min at room temperature before use. The successful siRNA loading was confirmed *via* agarose gel electrophoresis (**Supplementary Figure 1B**). A complete siRNA encapsulation was achieved at N/P > 2. In this study, N/P = 5 was used for the preparation of siRNA-loading NPs to avoid any undesired leaking. The resulting siRNA-loaded NPs could be efficiently taken up by cells. As monitored by fluorescence microscopy, significant cellular uptake was noted by RAW macrophage cells after co-culturing with FITC-labeled NPs for 16 h (**Supplementary Figure 1C**). The cytotoxicity of empty and siRNA loaded NPs was evaluated by MTT assay on RAW cells. Low cellular toxicity was observed at the concentration used for cellular uptake study (9.45 μg/ml, containing 100 pmol/ml siRNA) (**Supplementary Figure 1D**).

**Figure 1:**
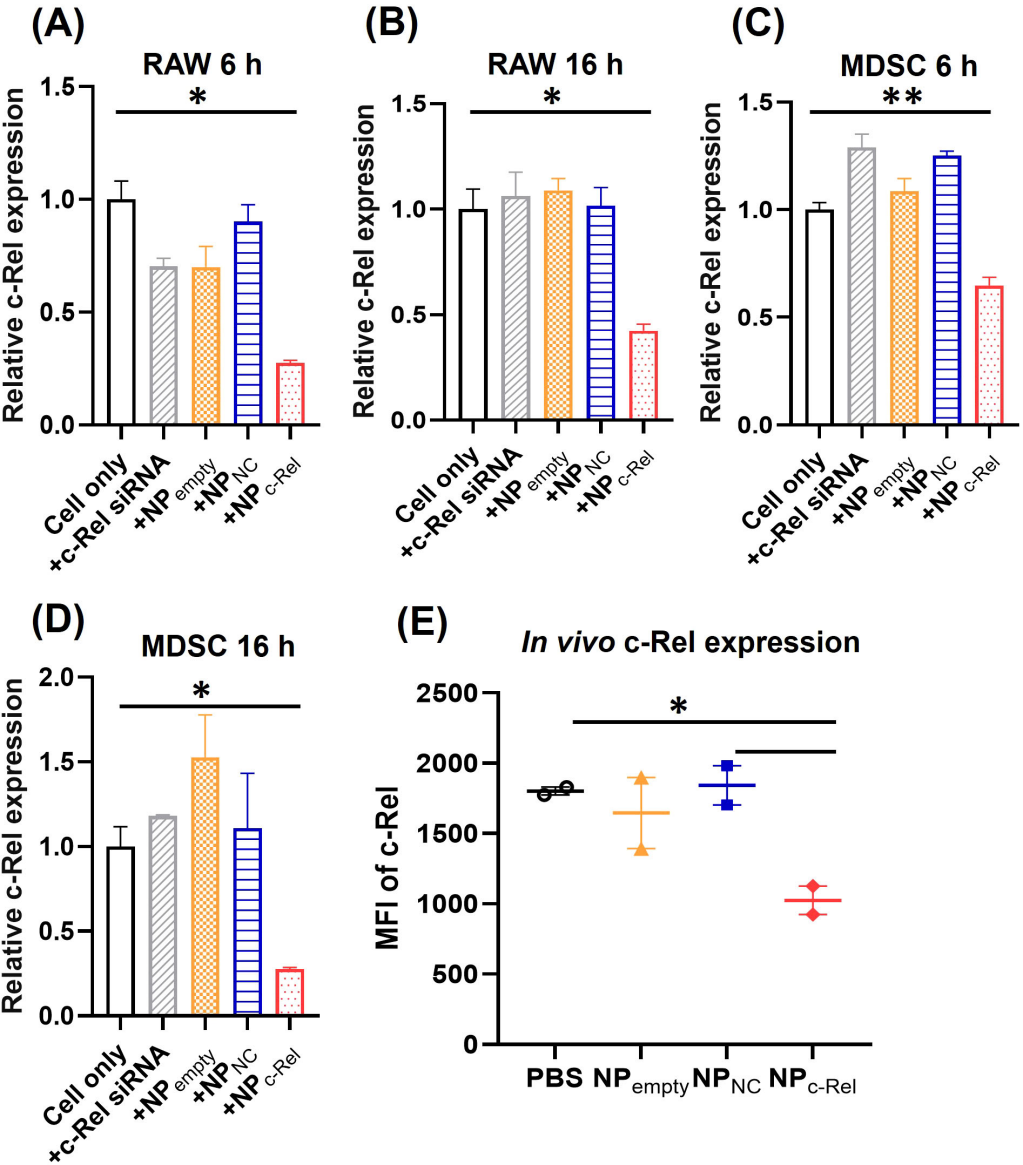
*In vitro* and *in vivo* c-Rel knockdown by c-Rel siRNA-loaded nanoparticles. **(A–D)** qPCR analysis of RAW cells **(A, B)** or bone marrow-derived MDSCs **(C, D)** incubated with vehicle control (Cell only), free c-Rel siRNA (c-Rel siRNA, 100 pmol/ml), c-Rel siRNA-loaded nanoparticles (NP_c-Rel_, containing 100 pmol/ml siRNA), negative control siRNA-loaded nanoparticles (NP_NC_, containing 100 pmol/ml siRNA) or empty nanoparticles (NP_empty_, containing equal amount of nanoparticles as NP_c-Rel_ and NP_NC_ groups) for 6 h **(A, C)** or 16 h **(B, D)**. **(E)** FACS analysis of c-Rel expression in blood circulating Gr-1^+^CD11b^+^ cells. Mice were i.v. injected with PBS, NP_c-Rel_ (2000 pmol siRNA), NP_NC_ (2000 pmol siRNA) or NP_empty_ (containing equal amount of nanoparticles as NP_c-Rel_ and NP_NC_ groups). Blood samples were collected 24 h post injection and stained with APC-anti-Gr-1, FITC-anti-CD11b and PE-anti-c-Rel antibody followed by FACS analysis of MFI-PE anti-c-Rel signal gated on Gr-1^+^CD11b^+^. Unpaired two-tailed Student’s *t* test was performed using Prism 10.0.3 (GraphPad) Software. *p<0.05, **p<0.01 [n = 3 for **(A–D)**, n = 2 for **(E)**]. **(A–D)** The results are representative of 3 independent experiments.

The *Rel* gene knockdown was achieved efficiently by the c-Rel siRNA-loaded nanoparticles *in vitro.* Compared to untreated cells, significant down-regulation of c-Rel mRNA expression (72.5%) was observed in RAW cells after 6 h incubation with NP_c-Rel_ based on qPCR analysis, and remained at a low level (58%) till 16 h after treatment (**Figures 1A, B**). Empty carriers (NP_empty_), non-encapsulated c-Rel siRNA and encapsulated negative control siRNA (NP_NC_) did not alter c-Rel mRNA level significantly. For *ex vivo* induced murine MDSCs, the c-Rel mRNA knockdown peak (73%) was observed after 16 h incubation with NP_c-Rel_, instead of 6 h for RAW cells, probably due to the slower cellular uptake of NPs by MDSCs compared to RAW cells. We next evaluated the protein expression level following c-Rel knockdown. The c-Rel protein expression was low in resting cells, and therefore, 50 ng/ml LPS was added to increase c-Rel expression in RAW cells. Compared to control groups, significantly reduced c-Rel protein expression (75%) was observed after 24 h incubation with NP_c-Rel_ (**Supplementary Figure 2A**). The aforementioned qPCR and Western blotting results indicated successful silencing of c-Rel expression by c-Rel siRNA-loaded nanoparticles *in vitro*.

We next evaluated the *in vivo* c-Rel knockdown efficiency of NP_c-Rel_. MDSCs in healthy mice are rare thus tumor-bearing mice were used. B16 tumor-bearing WT C57BL/6 mice were intravenously (i.v.) injected with PBS, empty NP, NP_NC_ (2000 pmol/mouse) or NP_c-Rel_ (2000 pmol/mouse). Blood sample was collected from tail vein 24 h after treatment. After red blood cell lysis, the cells were stained with antibodies against MDSC surface markers, Gr-1 and CD11b, then fixed and stained with anti-c-Rel antibodies for flow cytometry. As shown in **Figure 1E**, significant reduction (~50%) of c-Rel, as measured by mean fluorescence intensity (MFI), in NP_c-Rel_-treated mice was detected compared to other groups in CD11b^+^Gr-1^+^ myeloid cells, which indicated a strong *in vivo* knockdown. The knockdown of c-Rel expression can also be detected in spleen and lymph node samples 24 h after treatment, which indicated a successful systematic knockdown of c-Rel *in vivo* (**Supplementary Figure 2B**). The knockdown effect of c-Rel was weaker but still notable at 48 h, and disappeared 72 h post-treatment (**Supplementary Figures 2C, D**).

It is to be noted that the c-Rel knockdown effect mediated by the siRNA-loaded nanoparticles was selective for myeloid (CD11b^+^) cells since no significant c-Rel knockdown was observed for splenic CD4^+^ or CD8^+^ lymphocytes (**Supplementary Figures 2E–G**), which was likely due to the differences in phagocytic activity toward nanoparticles between lymphoid and myeloid cells.

### 3.2 Knocking down c-Rel by NP_c-Rel_ reduced the number and suppressive function of MDSCs *in vitro*

**Figure 2:**
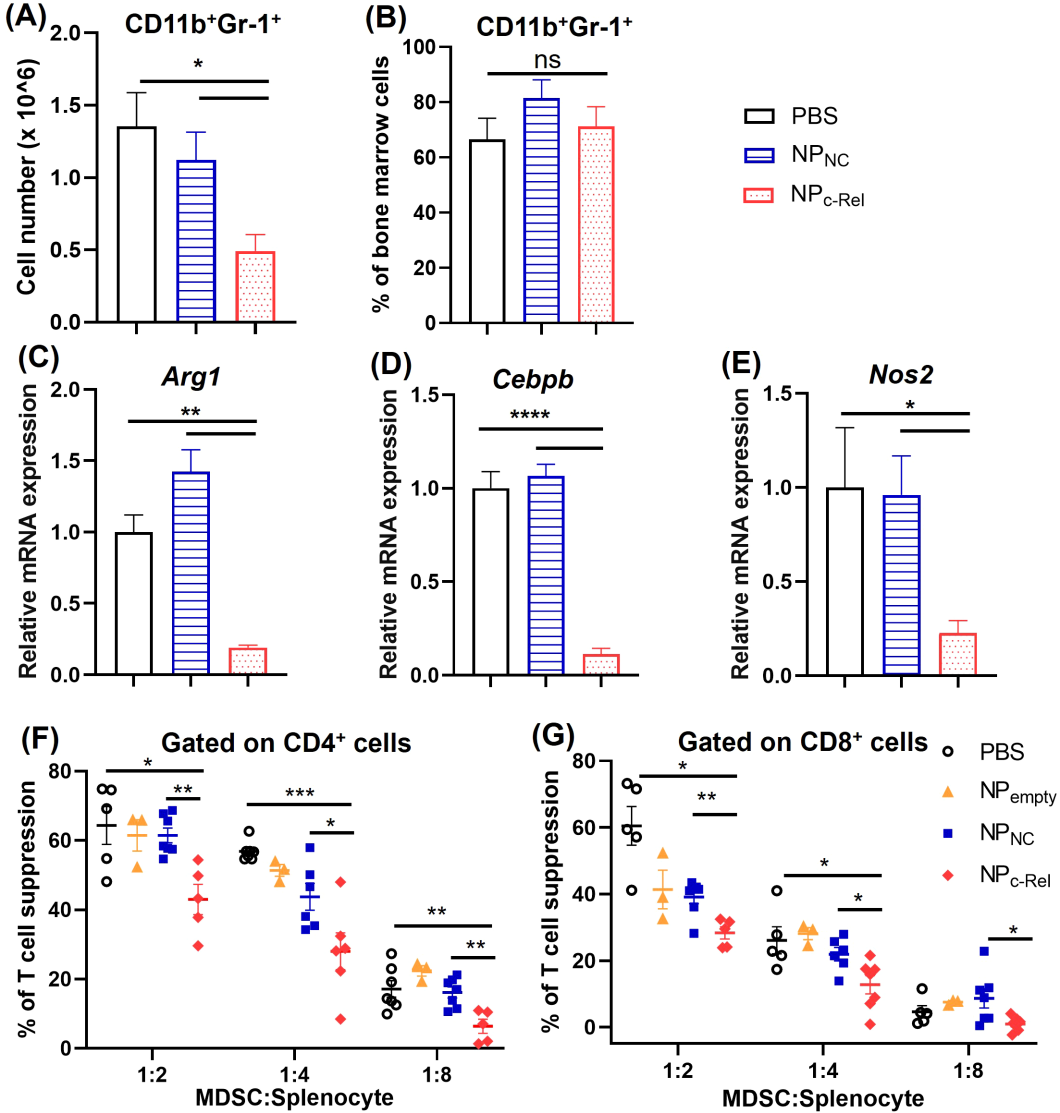
The effects of c-Rel knockdown on bone marrow-derived Gr-1^+^CD11b^+^ cells. 2 x 10^6^ bone marrow cells were isolated from mice and cultured with complete RPMI-1640 medium with GM-CSF (100 ng/ml) and IL-6 (100 ng/ml) for inducing the development of MDSCs. Cells were cultured for 5 days with PBS, NP_NC_ or NP_c-Rel_ (100 pmol/ml siRNA) added on day 0, day 2 and day 4. Cells were collected on day 5 for evaluation. **(A)** Gr-1^+^CD11b^+^ cell numbers per culture. **(B)** Percentages of Gr-1^+^CD11b^+^ cells in the culture as analyzed by flow cytometry. **(C**–**E)** qPCR analysis of *Arg-1*, *Cebpb* and *Nos2* mRNA expression in cultured cells. **(F, G)** T cell proliferation assay for the evaluation of immune suppressive functions of bone marrow-derived cells. Unpaired two-tailed Student’s *t* test was performed. ns, p>=0.05, *p<0.05, **p<0.01, ***p<0.001, ****p<0.0001. n = 3 for all groups in **(A–E)**; for **(F, G)**, n = 5 for PBS and NP_NC_ group, n = 6 for NP_c-Rel_, n = 3 for NP_empty_. **(A, B)** The results are representative of 3 independent experiments, **(C–E)** The results are representative of 2 independent experiments. **(F, G)** The results were pooled from 2 independent experiments.

After confirming the effective knockdown of c-Rel by NP_c-Rel_ *in vitro* and *in vivo*, we started to exam the effectiveness of c-Rel knockdown on MDSC development and function. Bone marrow cells containing myeloid progenitors isolated from healthy WT C57BL/6 mice were cultured in complete RPMI medium with IL-6 (100 ng/ml) and GM-CSF (100 ng/ml) for 5 days to induce the development of MDSCs. c-Rel siRNA-loaded particles (NP_c-Rel_), control siRNA-loaded particles (NP_NC_) or PBS vehicle control was added on day 0, 2 and 4 of the culture. By day 5, flow cytometry analysis indicated that c-Rel knockdown significantly reduced the expansion of the cultured cells. A significantly smaller Gr-1^+^CD11b^+^ myeloid cell population was observed in NP_c-Rel_ treated group compared to NP_NC_ and PBS group (0.49 ± 0.11x10^6^, 1.12 ± 0.19x10^6^, and 1.36 ± 0.23x10^6^ Gr-1^+^CD11b^+^ cells, respectively) (**Figures 2A, B**). MDSC signature genes such as *Arg1*, *Cebpb* and *Nos2* are crucial for maintaining their immune suppressive function. A significant down-regulation of MDSC signature genes was observed in NP_c-Rel_-treated group on day 5 by qPCR, while expression of these genes in the NP_NC-_ or PBS-treated cells remained high (**Figures 2C–E**). These results indicated that the knockdown of c-Rel in myeloid cells leads to a significant reduction of MDSC expansion and MDSC signature gene expression.

Further evaluation of MDSC function was done by evaluating their suppression of T cell proliferation *in vitro*. As described above, bone marrow cells from healthy WT C57BL6 mice were collected and cultured with IL-6 and GM-CSF for MDSC induction, with different treatments (NP_c-Rel_, NP_NC_ or PBS) used on day 0, 2 and 4. On day 5 of the culture, cells were collected and mixed with CFSE-labeled splenocytes isolated from healthy WT mice at different ratios (Gr-1^+^CD11b^+^ cells: splenocytes = 1:2, 1:4 and 1:8). Plate-bounded anti-CD3 and free anti-CD28 antibodies were used to induce the proliferation of T cells. Splenocytes cultured on the same plate without MDSCs served as controls. The cell mixtures were co-cultured for 48 h, then collected and stained with anti-mouse CD4 and anti-mouse CD8 antibodies followed by flow cytometry analysis. The proliferation of CD4^+^ or CD8^+^ T cells was evaluated by CFSE staining. We observed significantly reduced suppression of both CD4^+^ and CD8^+^ T cell proliferation by NP_c-Rel_-treated Gr-1^+^CD11b^+^ cells compared to NP_NC_ or PBS group (**Figures 2F, G**, **Supplementary Figures 3A–C**). These results indicated that the c-Rel knockdown via the c-Rel siRNA nanoparticles could significantly reduce the generation of MDSCs as well as diminish their immune suppressor functions.

### 3.3 Knocking down c-Rel suppressed tumor growth and reprogrammed tumor microenvironment *in vivo*

**Figure 3:**
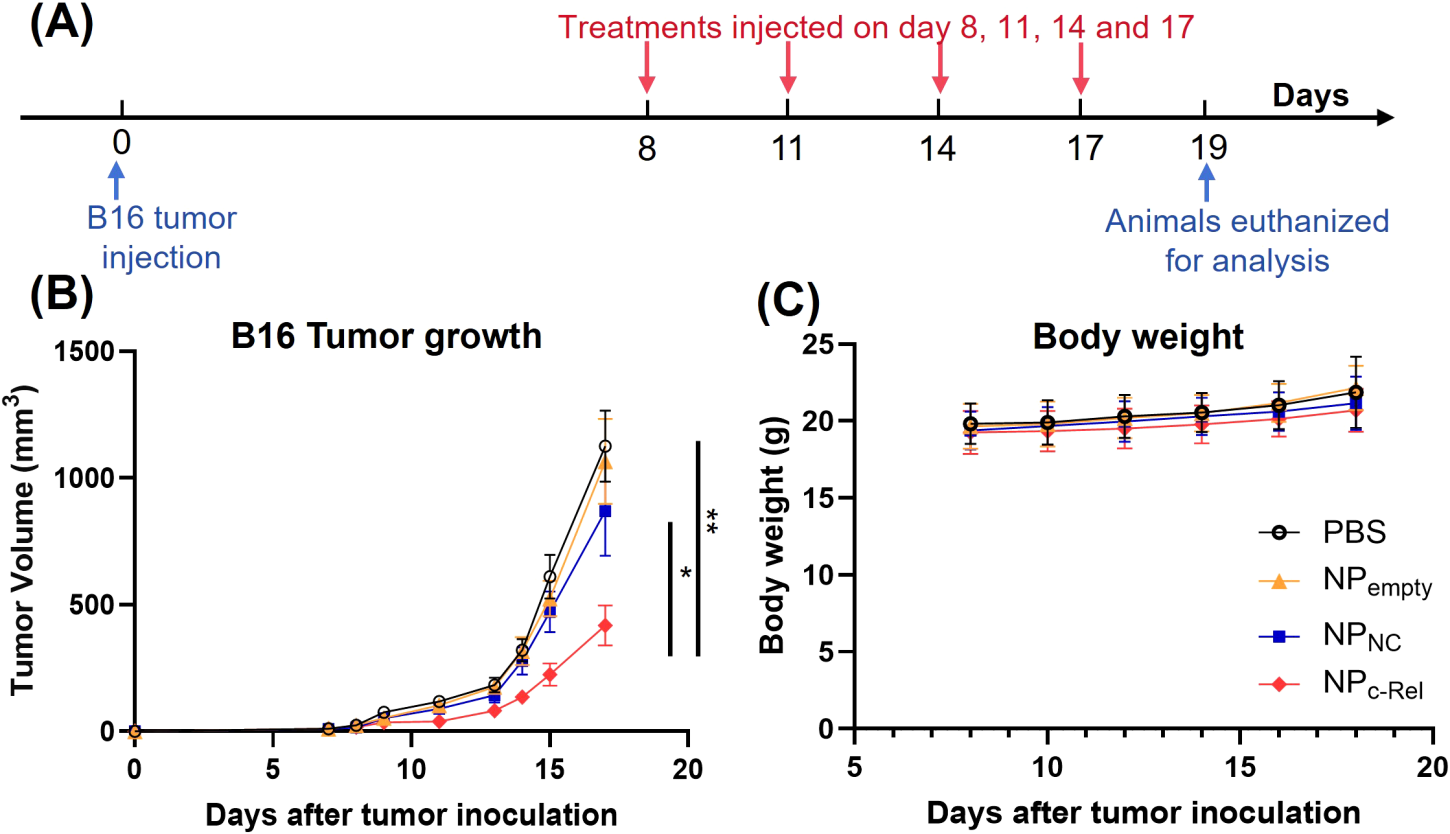
The effects of c-Rel knockdown on B16 tumor growth in mice. **(A)** Scheme of the tumor treatment schedule. Female C57BL/6 mice aged 6-8 weeks were injected with B16 tumor cells (1 x 10^5^) subcutaneously on day 0. Mice were randomized and divided into four groups on day 7. On day 8, 11, 14 and 17, mice received PBS, NP_c-Rel_ (2000 pmol siRNA/dose), NP_NC_ (2000 pmol siRNA/dose) or NP_empty_ (equal amount of polymer) through tail vein injections. Tumor growth and body weight were monitored every other day during the tumor challenge. Animals were euthanized for analysis on day 19. **(B)** B16 tumor growth curves. **(C)** Body weight changes of mice after tumor inoculation. Data were pooled from 3 independent experiments. PBS (n = 26 mice), NP_empty_ (n = 22 mice), NP_NC_ (n = 25 mice), NP_c-Rel_ (n = 30 mice). Unpaired two-tailed Student’s *t* test was performed. ns, not significant, *p<0.05, **p<0.01.

To test the therapeutic effect of NP_c-Rel_ *in vivo*, 6-8 week-old female WT C57BL/6 mice were subcutaneously inoculated with 1 x 10^5^ B16 tumor cells on their right flank on day 0. NP_c-Rel_ (2000 pmol siRNA/dose), NP_NC_ (2000 pmol siRNA/dose), NP_empty_ (equal amount of polymer) or PBS were administrated through tail vein on days 8, 11, 14 and 17 post tumor injection. The growth of tumors was monitored every other day. On day 19, animals were euthanized for further analysis (**Figure 3A**). No significant body weight loss was observed for mice received nanoparticles with or without siRNA (**Figure 3C**). The empty nanoparticle (NP_empty_) had negligible effect on B16 tumor growth. Encapsulation of control siRNA slightly reduced the tumor size as compared to the PBS group at 2000 pmol/dose (869 ± 176 vs 1125 ± 149 mm^3^, respectively), which was likely due to the immune adjuvant effect of the RNA in nanoparticles. A weak anti-tumor effect of NP_c-Rel_ was noted at 500 pmol/dose (**Supplementary Figure 4**), and more importantly, a significant reduction in tumor size (418 ± 78 mm^3^) was observed at 2000 pmol/dose of NP_c-Rel_ as compared to other groups (**Figure 3B**), indicating a strong and dose-dependent anti-tumor therapeutic effect of c-Rel siRNA-loaded nanoparticles.

We analyzed the numbers and percentages of immune cells in tumor and immune tissues by flow cytometry (**Supplementary Figure 5**). c-Rel knockdown using the c-Rel siRNA-loaded nanoparticles did not significantly change the numbers or percentages of total immune cells (CD45^+^), T helper cells (CD45^+^CD4^+^) or cytotoxic T cells (CD45^+^CD8^+^) when tumors of similar sizes in different treatment groups were analyzed by flow cytometry (**Supplementary Figures 6A–C, F–H**). The numbers and percentages of tumor-infiltrated suppressor cells such as MDSCs (CD11b^+^Gr-1^+^) and Tregs (CD45^+^CD4^+^FoxP3^+^) also remained at a similar level (**Supplementary Figures 6D, E, I, J**). We observed a decrease of Treg cells (CD45^+^CD4^+^FoxP3^+^) in tumor-draining lymph nodes (tdLN) in NP_c-Rel_-treated mice compared to PBS-treated mice; however, the percentages of the tdLN T helper cells (CD45^+^CD4^+^) and cytotoxic T cells (CD45^+^CD8^+^) were similar among all groups (**Supplementary Figures 7A, B, D**). The CD11b^+^Gr-1^+^ cells in tumor, tdLN, spleen and blood of the NP_c-Rel-_treated tumor-bearing mice stayed comparable to PBS and NP_empty_ groups (**Supplementary Figures 6D, I**, **Supplementary Figures 7C, E, F**). The NP_NC_ treatment did not change intra-tumor MDSC number or percentage (**Supplementary Figures 6D, I**), but reduced CD11b^+^Gr-1^+^ cell percentages in tdLNs, spleen and blood (**Supplementary Figures 7C, E, F**).

**Figure 4:**
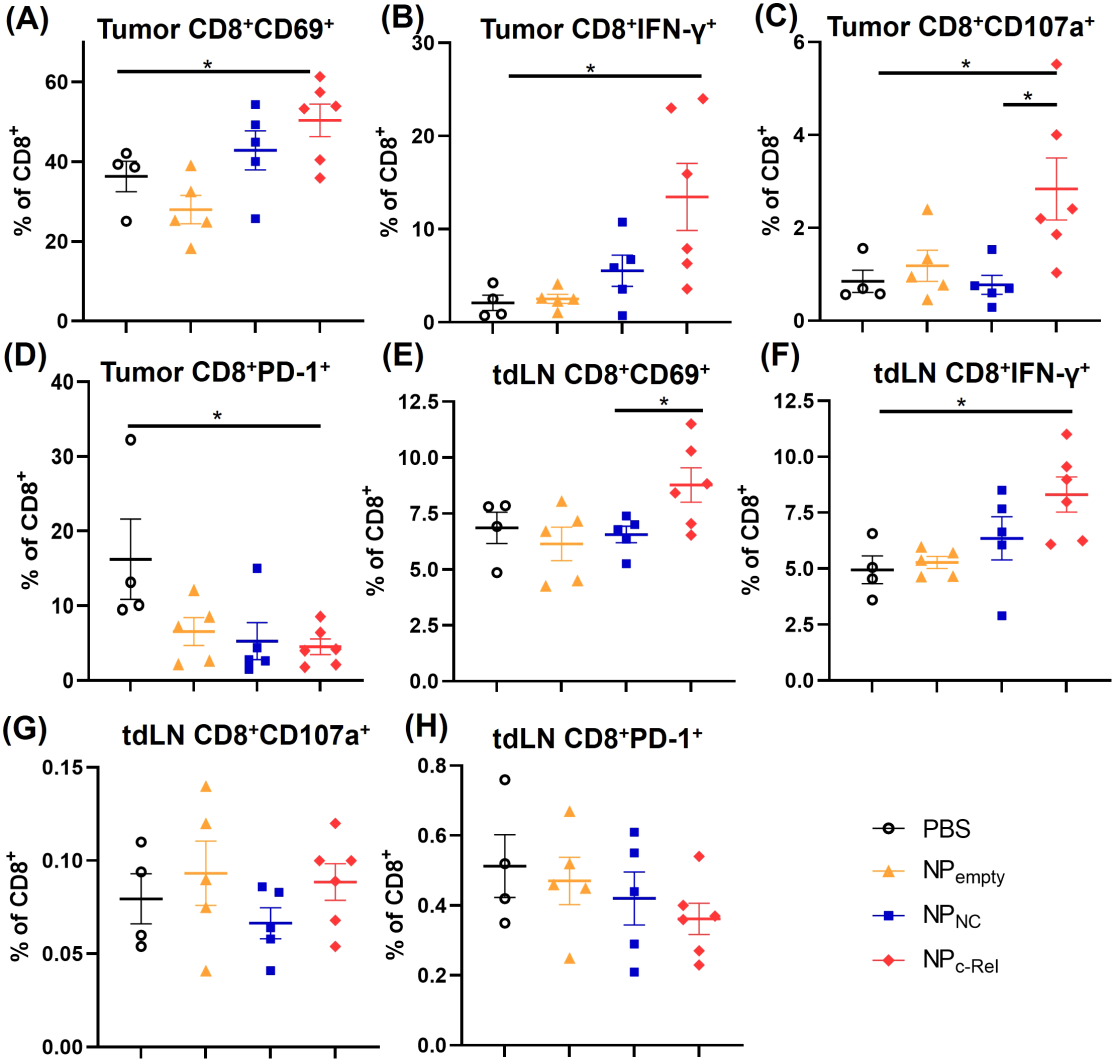
Characterization of CD8^+^ T cells in tumor-bearing mice. Mice were treated and sacrificed as described in **Figure 3**. Mice with similar tumor sizes from each treatment group were selected for this evaluation. PBS (n = 4 mice), NP_empty_ (n = 5 mice), NP_NC_ (n = 5 mice), NP_c-Rel_ (n = 6 mice). Tumors and tumor-draining lymph nodes (tdLN) were digested to prepare single cell suspensions. The percentages of intra-tumor CD8^+^CD69^+^ **(A)**, CD8^+^IFN-γ^+^ **(B)**, CD8^+^CD107a^+^ **(C)** and CD8^+^PD-1^+^ **(D)** cells, and tdLN CD8^+^CD69^+^ cells **(E)**, CD8^+^ IFN-γ^+^ **(F)**, CD8^+^CD107a^+^ **(G)** and CD8^+^PD-1^+^ **(H)** cells among CD45^+^CD8^+^ cells were determined by flow cytometry. Unpaired Student’s *t* test was performed, *p<0.05.

Although c-Rel knockdown did not significantly change the numbers and frequencies of immune cells, it did significantly increase the activities of cytotoxic T cells in the tumor. Flow cytometric analysis showed a significant increase in the percentages of CD8^+^IFN-γ^+^, CD8^+^CD69^+^ and CD8^+^CD107a^+^ cells in NP_c-Rel_-treated tumors compared to the PBS group (**Figures 4A–C**, **Supplementary Figure 8**), while the percentages of PD-1^+^CD8^+^ T cells were reduced (**Figure 4D**, **Supplementary Figure 8**), indicating a notable enhancement of CD8^+^ T cell activation. In tdLNs, NP_c-Rel_ treatment also increased IFN-γ^+^ and CD69^+^ CD8^+^ T cells, but not CD107a^+^ or PD-1^+^ CD8^+^ T cells (**Figures 4E–H**). In contrast, neither NP_NC_ nor NP_empty_ increased T cell activation, either in the tumor or tdLN as compared to PBS (**Figures 4A–H**).

### 3.4 Knocking down c-Rel reduced the suppressive function of MDSCs in mice

**Figure 5:**
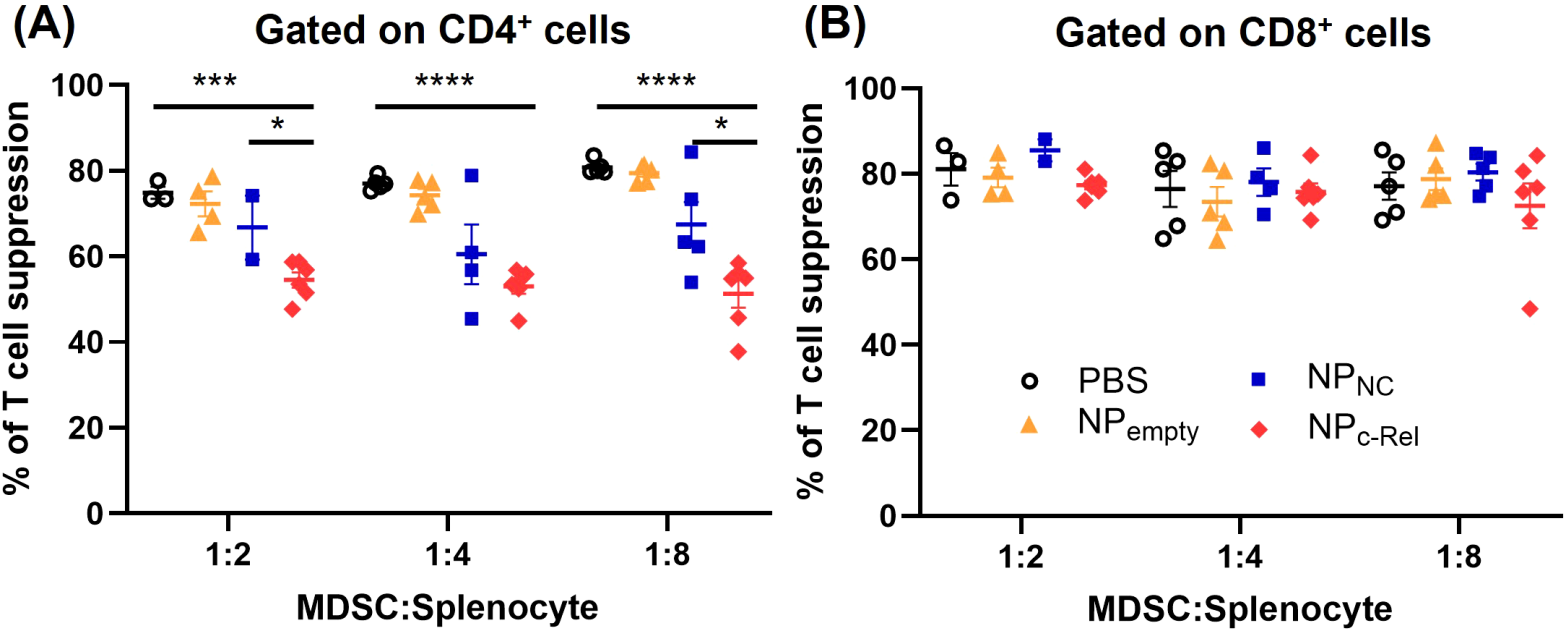
Immune suppression evaluation of Gr-1^+^CD11b^+^ cells isolated from the spleens of tumor-bearing mice. Mice in **Figure 4** were used for isolation of splenic MDSCs [PBS (n = 4 mice), NP_empty_ (n = 5 mice), NP_NC_ (n = 5 mice), NP_c-Rel_ (n = 6 mice)]. T cell suppression assay was performed as described in Methods at MDSC: Splenocyte ratios of 1:2, 1:4 and 1:8 for CD4^+^ **(A)** and CD8^+^ **(B)** splenocytes. Unpaired two-tailed Student’s *t* test was performed, *p<0.05, ***p<0.001, ****p<0.0001. Some data points in MDSC: Splenocyte = 1:2 and 1:4 groups were not available due to the limited amount of MDSCs isolated from the corresponding mice.

Although the CD11b^+^Gr-1^+^ cell numbers in NP_c-Rel_-treated mice remained similar to those in the PBS-, NP_NC_- or NP_empty_-treated mice, we wondered whether their suppressive function was affected by the c-Rel knockdown. Splenic CD11b^+^Gr-1^+^ cells were isolated using negative magnetic cell separation (MACS) kits from mice bearing tumors of similar sizes in each treatment groups (to minimize the impact of tumor sizes), and their immune suppressive function evaluated in the T cell proliferation assay as described in Methods. We found that CD11b^+^Gr-1^+^ cells isolated from PBS- or NP_empty_-treated tumor-bearing mice strongly suppressed both CD4^+^ and CD8^+^ T cell proliferation (74.86-80.78% for CD4^+^ T cells, 76.64 -81.1% for CD8^+^ T cells) (**Figure 5**, **Supplementary Figure 9**). c-Rel knockdown by NP_c-Rel_ significantly reduced the splenic MDSC suppressive activity on CD4^+^ T cells as compared to PBS-treated group (51.30-54.52%) (**Figure 5A**, **Supplementary Figure 9A**), but had no detectable effect on their suppression on CD8^+^ T cells (72.53-77.34%) (**Figure 5B**, **Supplementary Figure 9B**). Unlike MDSCs induced *in vitro* shown in **Figures 2F, G**, these MDSCs isolated from tumor-bearing mice showed a selective effect on different T cell subsets, likely due to the differences in MDSC development stage and/or microenvironment.

## 4 Discussion

Immune checkpoint therapy has revolutionized cancer treatment in the clinic. Several immune checkpoint inhibitor drugs have been approved by food and drug administration (FDA) covering multiple cancer types (2, 4, 24). Although improved clinical efficacy has been reported, the current immune checkpoint therapy only benefits a small proportion of cancer patients. Patients who initially respond to the therapy may also acquire resistance during the course of the treatment (1, 3, 4). The current FDA-approved immune checkpoint inhibitor drugs including those for CTLA-4, PD-1 and PD-L1 target mainly lymphoid cells, whereas checkpoint therapy drugs against myeloid cells were less investigated. Since myeloid cells also play important roles in generating the immune-suppressive microenvironment of tumor tissues, developing myeloid checkpoint drugs may provide an alternative for patients who are not responding to, or have developed resistance against, the current lymphoid checkpoint therapies (25–27).

Although c-Rel was previously known as an inflammatory factor and therapeutic target for inflammatory diseases such as psoriasis and arthritis, its anti-tumoral effect was less well evaluated. In this work, we found that c-Rel siRNA-loaded particles, NP_c-Rel_, successfully knocked down c-Rel expression *in vitro* and *in vivo*, and conferred a significant anti-tumor effect in mice. Along with the reduced tumor growth, the reprogramming of tumor microenvironment was also observed in NP_c-Rel_-treated mice. Though the tumor-infiltrating immune cell numbers tend to remain unchanged, the anti-tumor immune activity of tumor-infiltrating CD8^+^ T cells was significantly enhanced by the c-Rel knockdown. Significantly higher percentages of CD69^+^, CD107a^+^ and IFN-γ^+^ tumor-infiltrating CD8^+^ T were detected in NP_c-Rel_-treated mice compared to the PBS group. By contrast, the PD-1^+^ CD8^+^ T cell percentages were reduced after NP_c-Rel_ treatment, suggesting a reduced exhaustion of tumor-infiltrating CD8^+^ T cells. The MDSCs isolated from NP_c-Rel_ treated mice, when tested *in vitro*, significantly reduced the suppression of CD4^+^ T cells, which are crucial for CD8^+^ T cell activation. Therefore, we purpose this enhanced CD8^+^ T cell function could be indirectly contributed by the enhanced CD4 T cell function when the suppressive activity of MDSCs is compromised by NP_c-Rel_ therapy.

MDSCs are one of the most important immune suppressive cell types in tumor microenvironment, and c-Rel is an important promoter for MDSC development. In this work, we found that knocking down c-Rel by NP_c-Rel_ reduced MDSC numbers and inhibited the expression of MDSC signature genes such as *Arg-1*, *Cebpb* and *Nos2* of Gr-1^+^CD11b^+^ cells *in vitro.* The c-Rel knockdown also significantly diminished the suppressive function of Gr-1^+^CD11b^+^ cells in the T cell proliferation assay. Therefore, unlike the lymphoid immune checkpoint drugs such as PD-1 antibodies that directly target CD8^+^ T lymphocytes, the control of tumor growth and CD8^+^ T activity by c-Rel knockdown is more likely contributed by the diminished MDSC function and the resulting changes in the immune suppressive tumor microenvironment.

In summary, we found that c-Rel expression in myeloid cells can be successfully knocked down via c-Rel siRNA-loaded nanoparticles in mice, which can significantly diminish tumor growth by reprograming the immunosuppressive tumor microenvironment. Therefore, targeting myeloid c-Rel can be an effective new strategy for the treatment of cancer.

## Data Availability

The raw data supporting the conclusions of this article will be made available by the authors, without undue reservation.
