# Supplementary material for: Cancer immunotherapy by silencing transcription factor c-Rel using peptide-based nanoparticles
Source: Front Immunol. 2025 Mar 11;16:1554496. doi: 10.3389/fimmu.2025.1554496 (PMC11933105; doi:10.3389/fimmu.2025.1554496)
Supplement: Supplementary file 1 [file DataSheet1.docx]

***Supplementary Material***


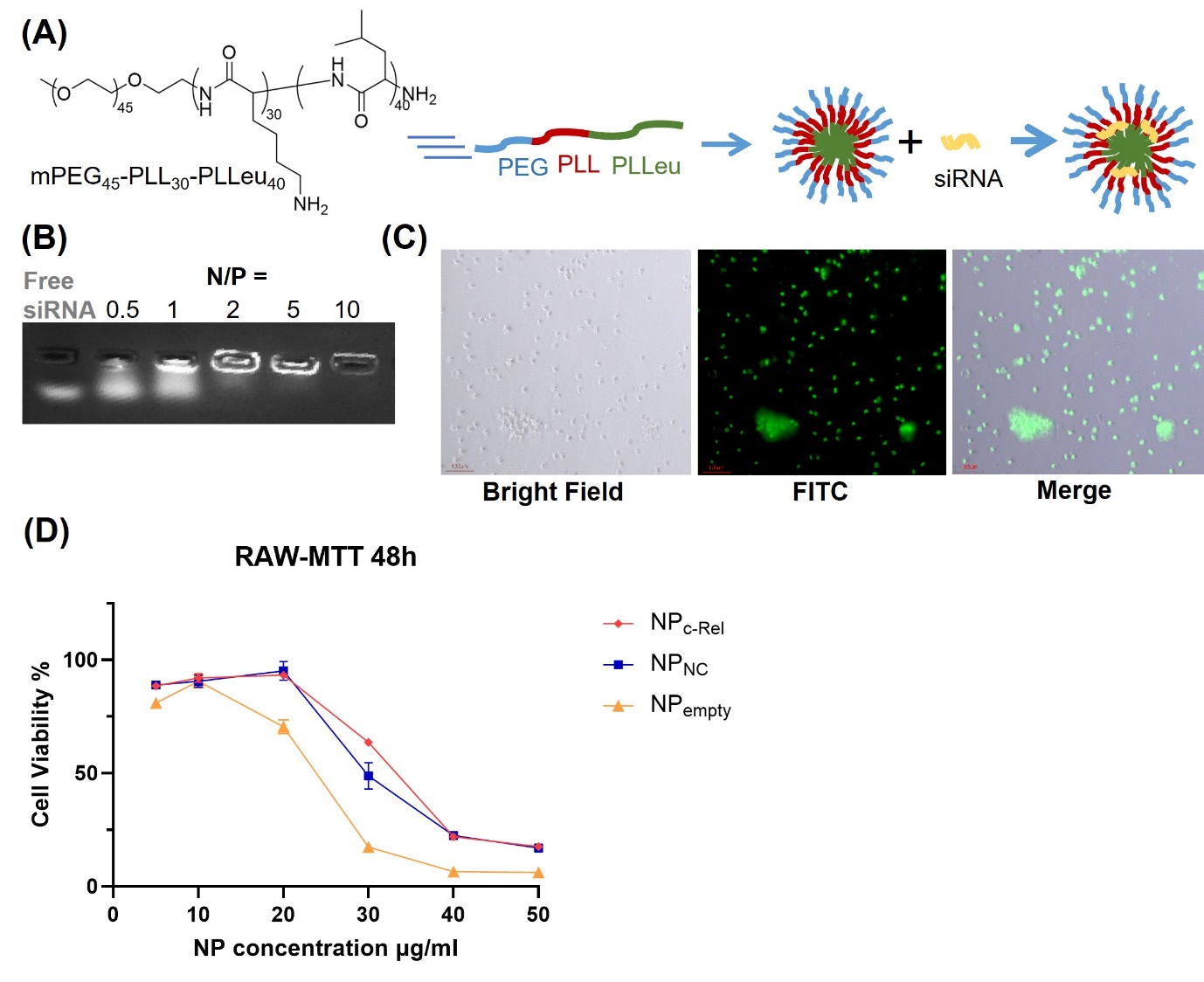


**Supplementary Figure 1.** c-Rel siRNA encapsulation and cell uptake. **(A)** Scheme of mPEG_45_-PLL_30_-PLLeu_40_ nanoparticle preparation and siRNA loading; **(B**) Agarose gel electrophoresis to confirm the encapsulation of siRNA. Complete encapsulation achieved at N/P>=2. In this paper, N/P=5 was used for *in vitro* and *in vivo* experiment. **(C)** RAW cells were co-cultured with FITC-labeled mPEG_45_-PLL_30_-PLLeu_40_ for 16 h and were then observed with fluorescence microscope. **(D)** Cellular toxicity of NP_c-Rel_, NP_NC_ and NP_empty_ on RAW cells at different NP concentrations (5, 10, 20, 30, 40, 50 μg/ml). MTT assay were performed after 48 h co-incubation for cell viability evaluation.


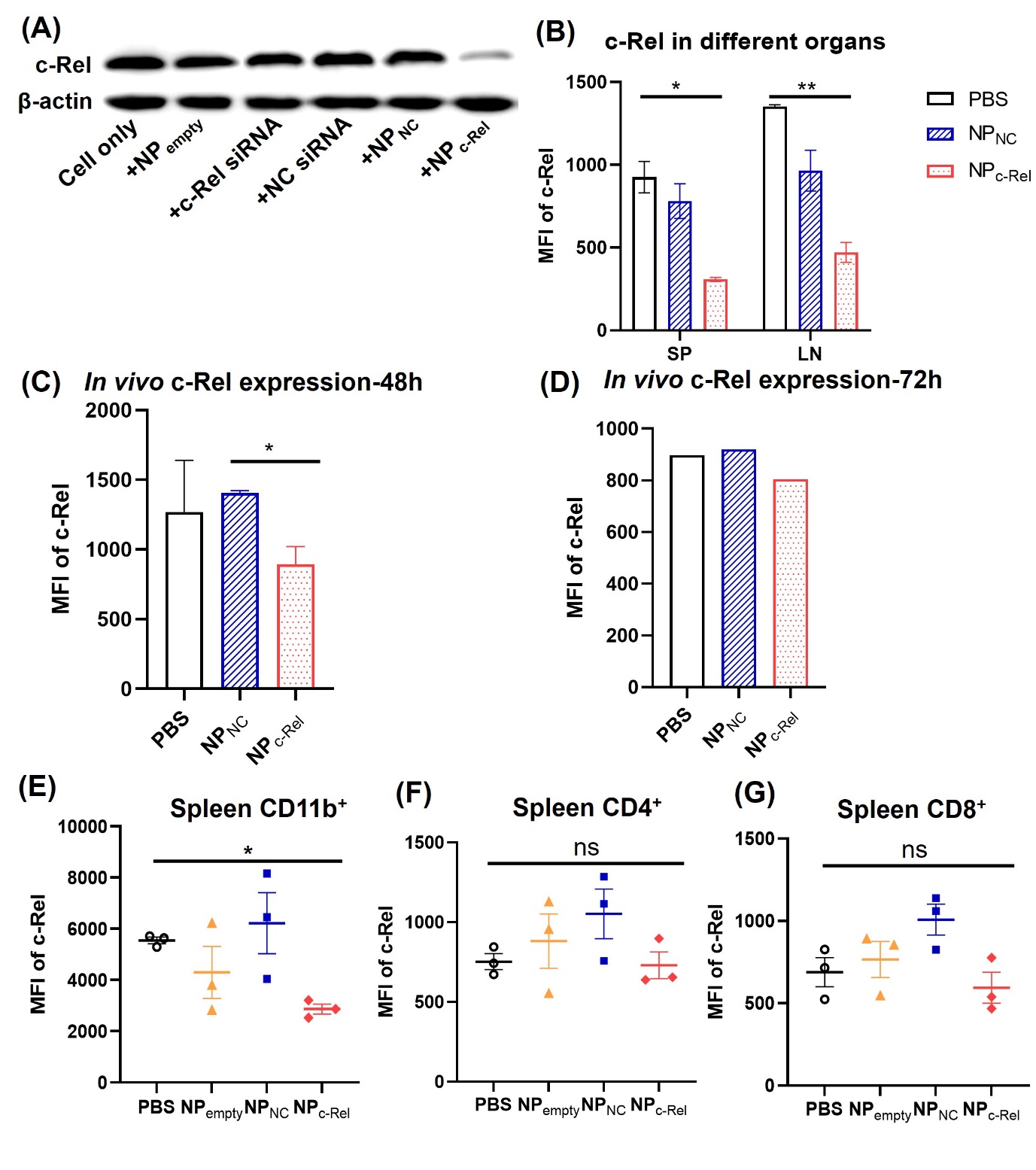


**Supplementary Figure 2.** *In vitro* and *in vivo* c-Rel knockdown. **(A)** Western blot detection of c-Rel protein in RAW cells with or without the indicated treatment for 24h. 50 ng/ml LPS was also added to the cultures to induce c-Rel expression. **(B)** *In vivo* c-Rel expression in different organs (SP: Spleen, LN: Lymph node) 24 h after i.v. injection of PBS, NP_NC_ (2000 pmol) or NP_c-Rel_ (2000 pmol). MFI of c-Rel was measured in CD45^+^CD11b^+^ cells. (**C-D**) *In vivo* c-Rel expression in blood 48 h (**C**) and 72 h (**D**) after i.v. injection of PBS, NP_NC_ (2000 pmol) or NP_c-Rel_ (2000 pmol). MFI of c-Rel was measured in CD45^+^CD11b^+^ cells. **(E-G)** c-Rel knockdown in splenocyte subsets after the indicated treatments for 24h *ex vivo*. MFI of c-Rel in splenic CD11b^+^ **(E)**, CD4^+^ **(F)** and CD8^+^ **(G)** cells was measured by flow cytometry. Unpaired two-tailed Students *t* test was performed. ns: p>=0.05, *p<0.05, **p<0.01. (n = 2 for **B-D**, n = 3 for **C**-**E**)


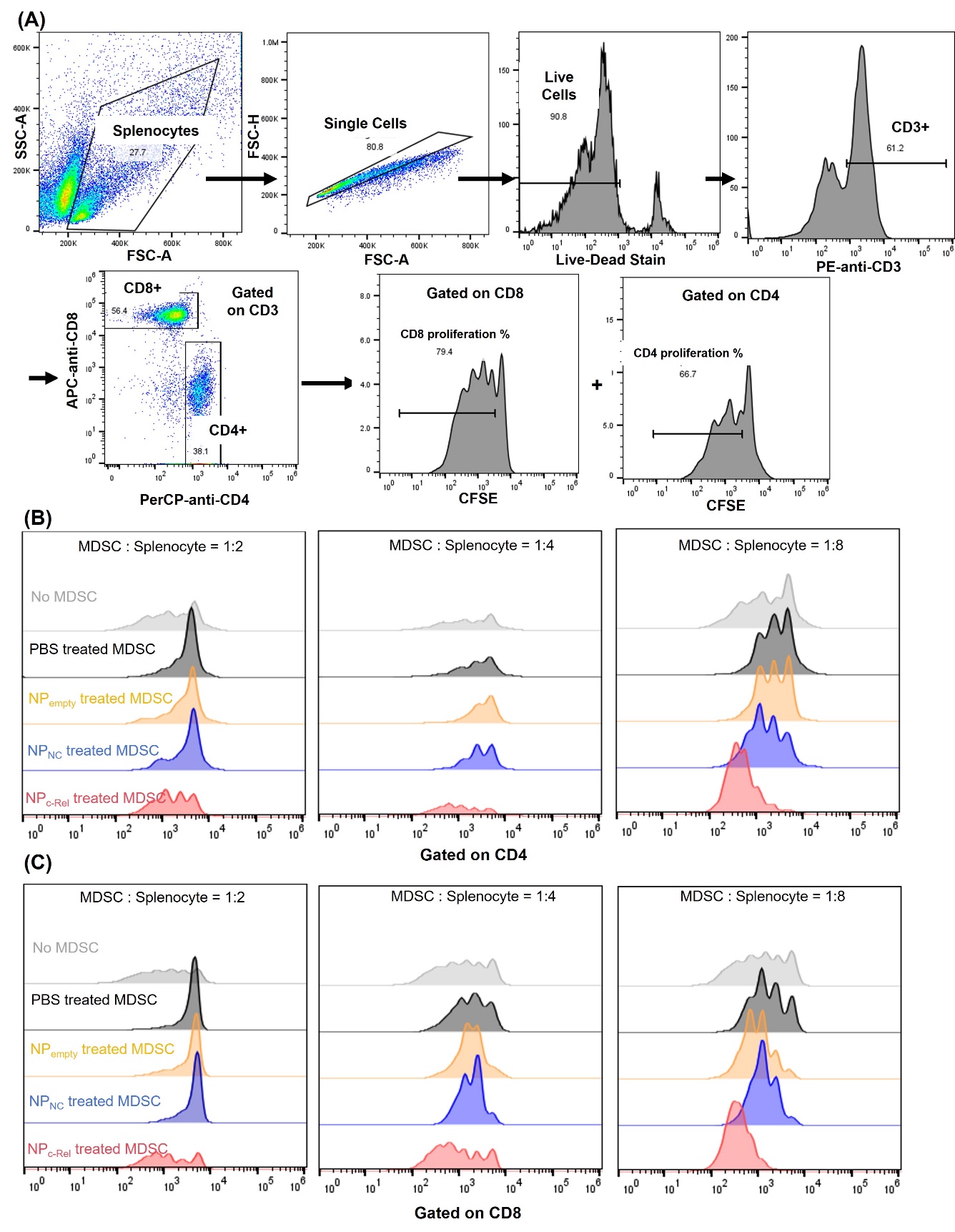


**Supplementary Figure 3.** Scheme of flow cytometry gating and representative data of the T cell proliferation assay presented in **Figure 2F-G**. **(A)** Scheme of flow cytometry gating for the measurement of T cell proliferation percentage. **(B-C)** Representative flow cytometry data of CD4^+^ T **(B)** and CD8^+^ T cell **(C)** proliferation when co-cultured with bone marrow-derived MDSCs. The summarized data and the statistical analysis were presented in **Figure 2F-G**.


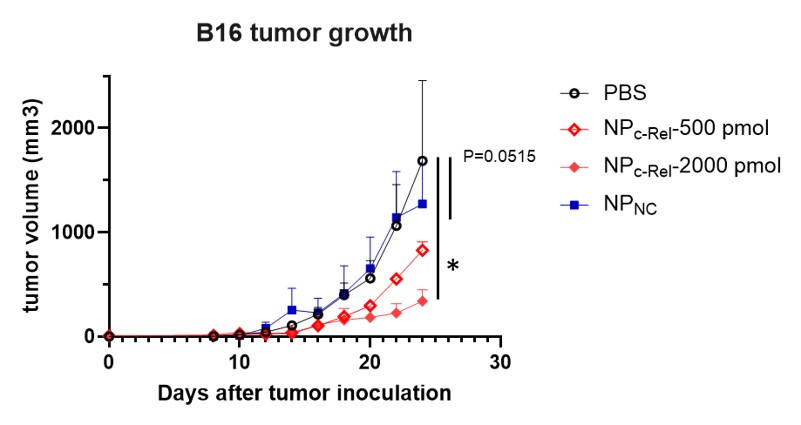


**Supplementary Figure 4.** The dose-dependent effect of c-Rel knockdown on B16 tumor growth in mice. Female C57BL/6 mice aged 6-8 weeks were injected with B16 tumor cells (1 x 10^5^) subcutaneously on day 0. Mice were randomized and divided into four groups on day 7. On day 8, 11, 14 and 17, mice received PBS, NP_c-Rel_ (500 pmol siRNA/dose), NP_c-Rel_ (2000 pmol siRNA/dose) or NP_NC_ (2000 pmol siRNA/dose) through tail vein injections. Tumor growth and body weight were monitored every other day during the tumor challenge.


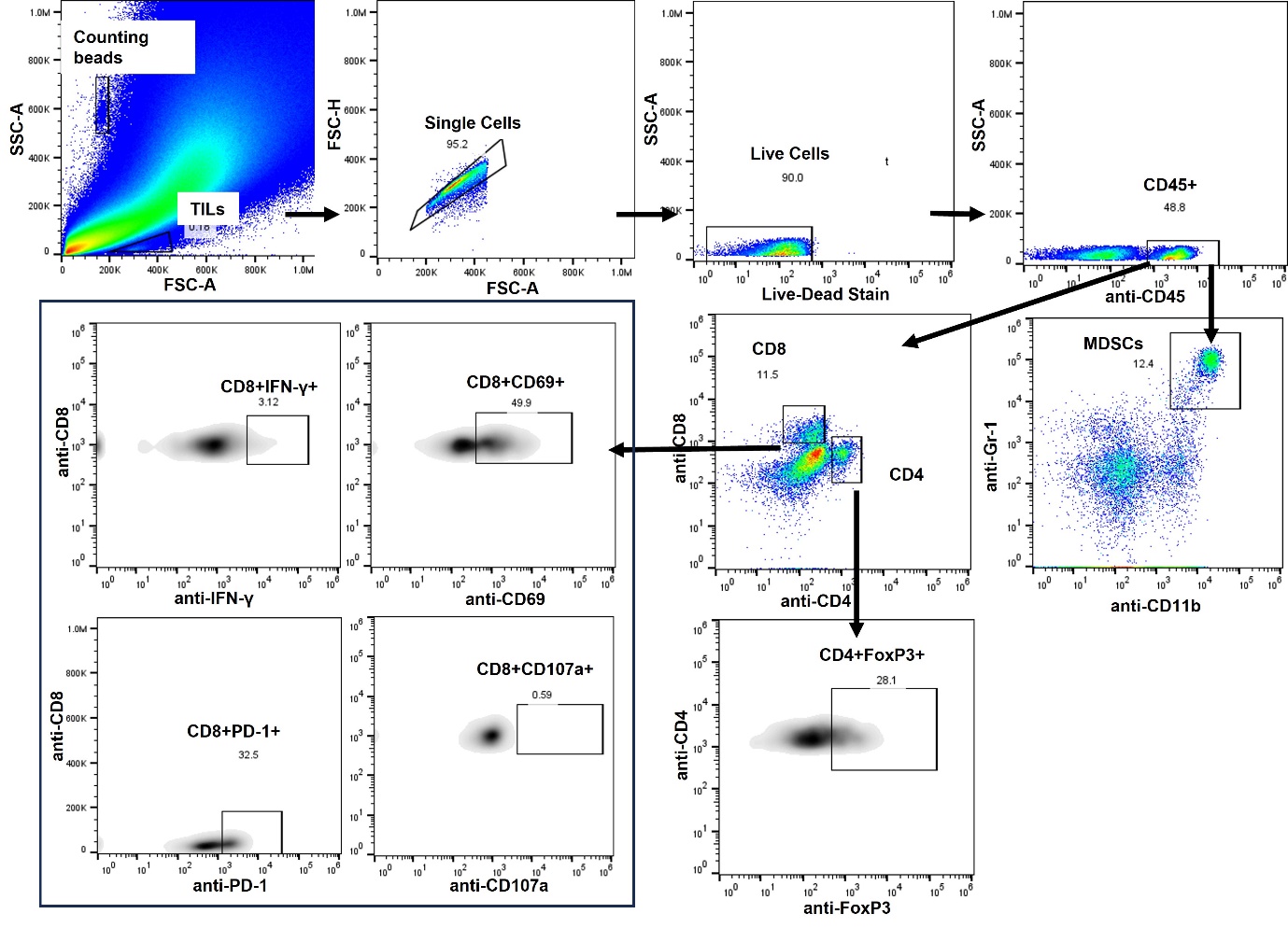


**Supplementary Figure 5.** Scheme of flow cytometry gating for the analysis of tumor infiltrated immune cells. Mice were treated as in **Figure 3**, and those with similar tumor sizes from each group were sacrificed on Day 19 and used for this analysis. The numbers, percentages and activation markers of the tumor-infiltrated immune cells were gated as described in figure. The numbers of immune cells per gram tissue were calculated based on the number of flow cytometry counting beads and tumor weight. Summarized data were presented in **Figure 4** and **Supplementary Figure 6**. The immune cell numbers and percentages in spleen, blood and lymphoid tissues were analyzed by the same gating strategy. Summarized data were presented in **Supplementary Figure 7**.


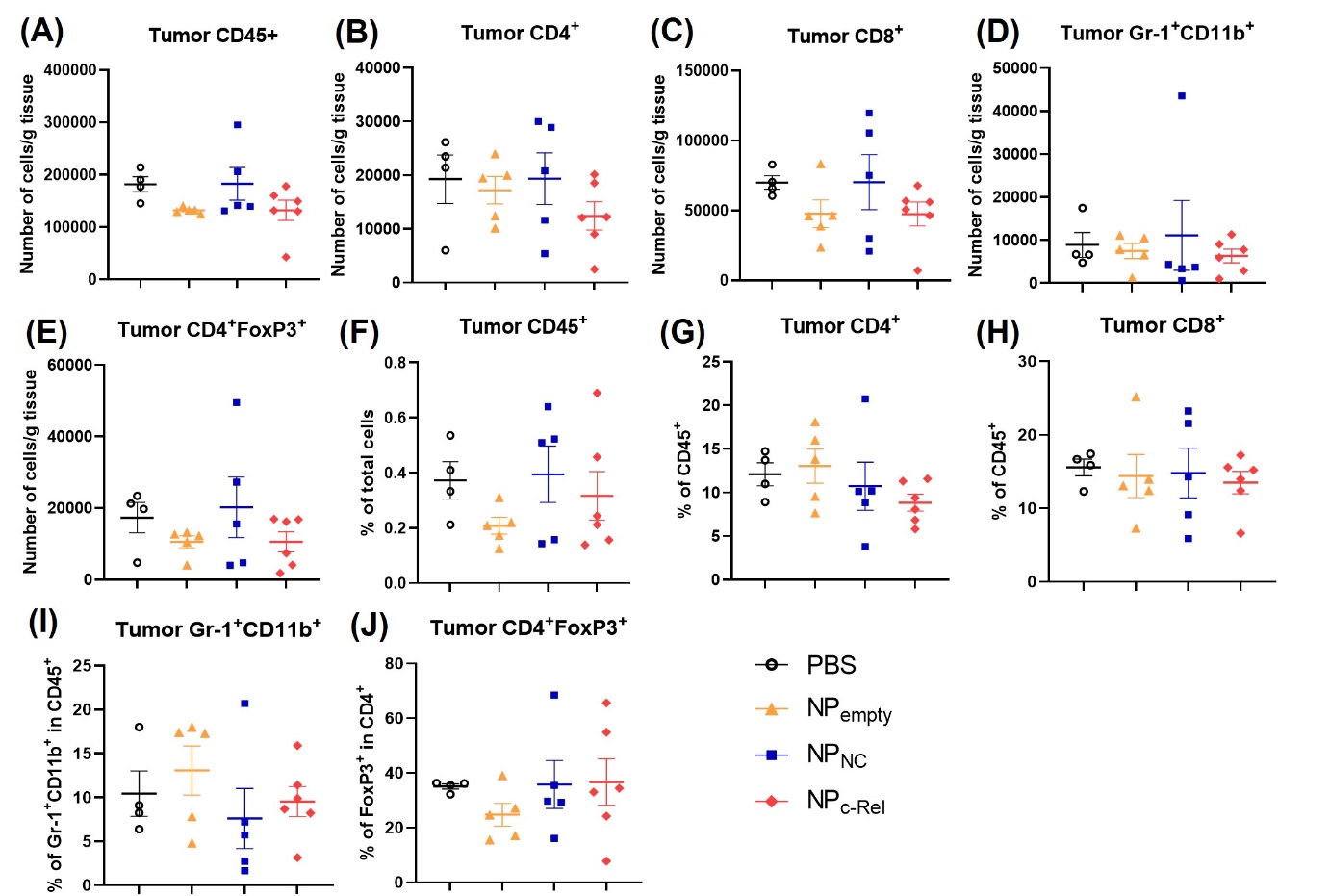


**Supplementary Figure 6**. Immune cell numbers and percentages in tumors. Mice were treated as in **Figure 3**, and those with similar tumor sizes from each group were sacrificed on Day 19 and used for this analysis. Numbers of tumor-infiltrating CD45^+^ **(A)**, CD4^+^ **(B)** , CD8^+^ **(C)**, Gr-1^+^CD11b^+^ **(D)** and CD4^+^Foxp3^+^ **(E)** cells per gram of tumor tissues, as well as the percentages of tumor-infiltrating CD45^+^ cells **(F)** in all cells collected, CD4^+^ **(G)**, CD8^+^ **(H)** and Gr-1^+^CD11b^+^ cells **(I)** in CD45^+^ cells, and CD4^+^Foxp3^+^ cells **(J)** in CD45^+^CD4^+^ cells were determined by flow cytometry. PBS (n = 4), NP_empty_ (n = 5), NP_NC_ (n = 5), NP_c-Rel_ (n = 6). Unpaired two-tailed Students *t* test was performed; no significant differences (p<0.05) were noticed between groups.N=3 for all groups. Unpaired two-tailed Students’ t test was performed, *p<0.05.


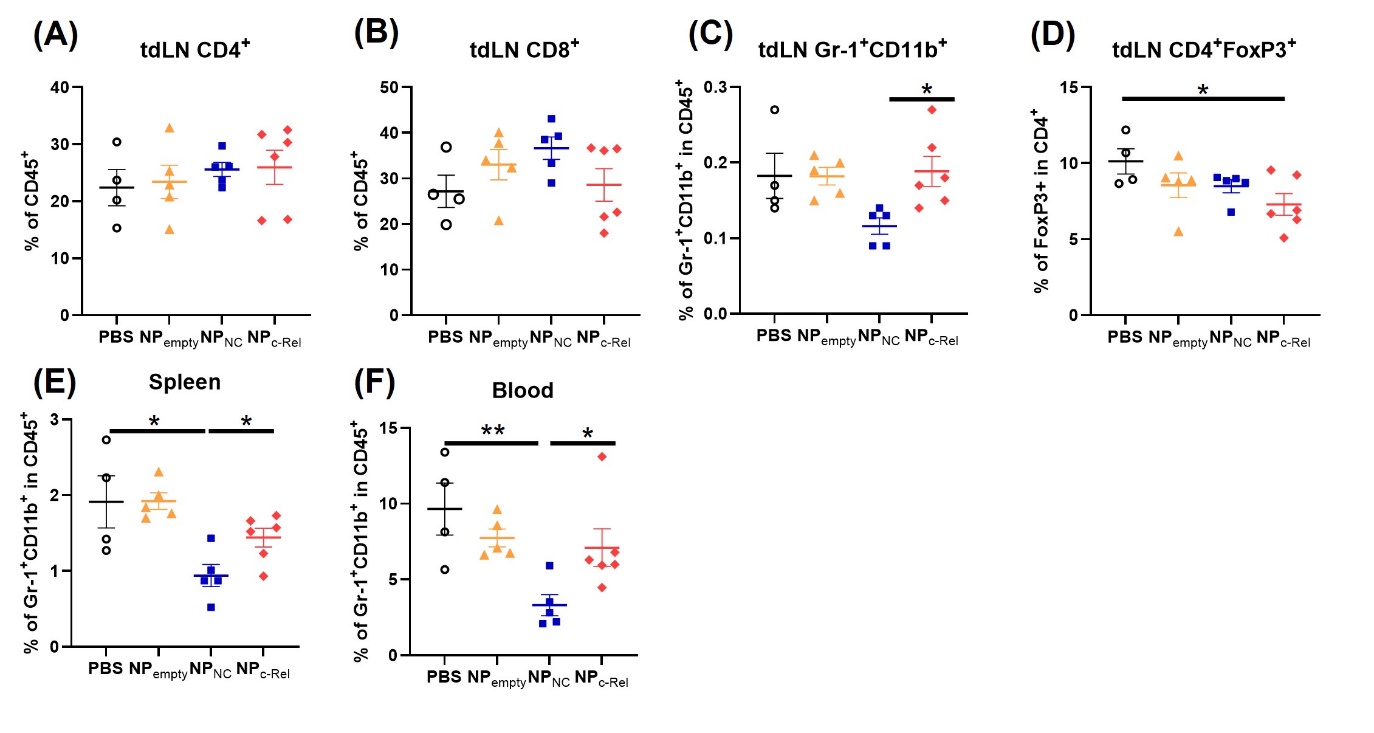


**Supplementary Figure 7.** Immune cell numbers and percentages in spleen, blood and lymphoid tissues. Mice were treated as in **Figure 3**, and those with similar tumor sizes from each group were sacrificed on Day 19 and used for this analysis. Tumor draining lymph nodes (tdLNs), spleen and blood were collected for flow cytometric analyses. Percentages of CD4^+^ cells **(A)**, CD8^+^ cells **(B)**, Gr-1^+^CD11b^+^ cells **(C)** and CD4^+^Foxp3^+^ cells **(D)** in CD45^+^ tdLNs cells, and percentages of Gr-1^+^CD11b^+^ cells in spleen **(E)** and blood **(F)** CD45^+^ cells were shown. PBS (n = 4), NP_empty_ (n = 5), NP_NC_ (n = 5), NP_c-Rel_ (n = 6). Unpaired two-tailed T test was performed, *p<0.05, **p<0.01.

**
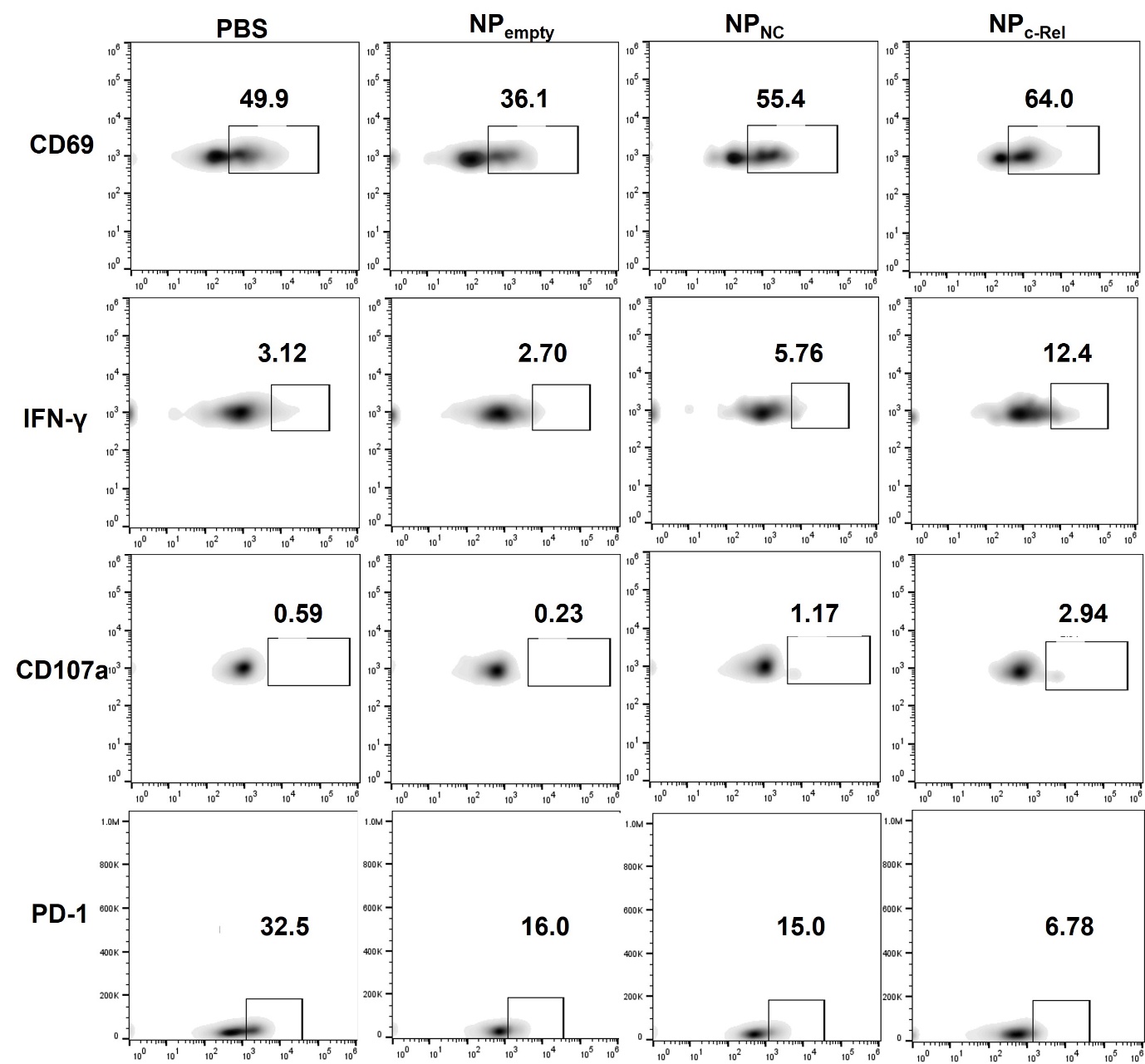
**

**Supplementary Figure 8.** Representative flow cytometry images of the characterization of CD8^+^ T cells in tumor-bearing mice. Summarized data was presented in **Figure 4**.

**
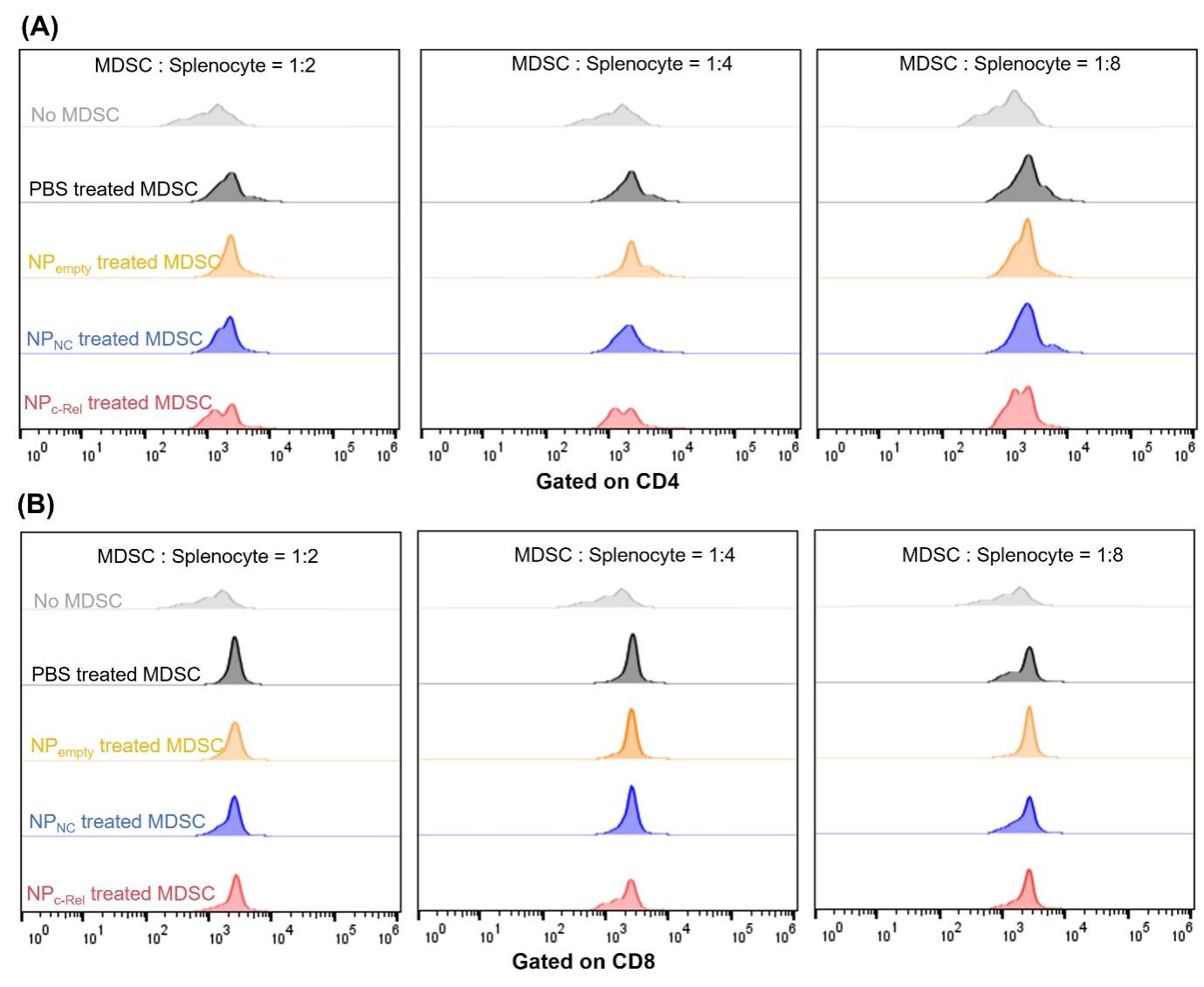
**

**Supplementary Figure 9.** Representative flow cytometry data of CD4^+^ T **(A)** and CD8^+^ T cell **(B)** proliferation when co-cultured with MDSCs isolated from the spleen of tumor-bearing mice. The summarized data and the statistical analysis were presented in **Figure 5**.
